# Dehydrocorydaline Exerts Anti-Pancreatic Cancer Effects Through the PI3K/Akt/mTOR Pathway

**DOI:** 10.3390/ph19060864

**Published:** 2026-05-29

**Authors:** Qingmeng Yu, Ruiding Li, Zhengyu Li, Zhexin Wan, Kaikai Lv, Chongyang He, Jianfang Sun, Shubing Jia, Yijia Xu, Mingyi Zhao

**Affiliations:** 1 School of Clinical Pharmacy, Shenyang Pharmaceutical University, Shenyang 110016, China; m1371052024@163.com (Q.Y.); 18563827779@163.com (Z.W.); hechongyang2022@163.com (C.H.); jiashubing1023@163.com (S.J.); 2 School of Life Sciences and Biopharmaceutical Science, Shenyang Pharmaceutical University, Shenyang 110016, China; lrd0511@163.com (R.L.); zhengyuli0421@163.com (Z.L.); sunjianfang0710@126.com (J.S.)

**Keywords:** dehydrocorydaline, pancreatic cancer, signal pathway, anti-tumor, key target

## Abstract

**Objectives:** This study aims to investigate the pharmacological effects and potential mechanisms of dehydrocorydaline, the primary active component of *Corydalis yanhusuo* W.T. Wang, as a potential therapeutic agent for pancreatic cancer, thereby providing new insights into its treatment. **Methods:** The pharmacological effects were assessed through MTT assay, colony formation assay, flow cytometry, scratch wound assay, and transwell assay. Potential mechanisms were explored through bioinformatics analysis and Western blot. **Results:** Dehydrocorydaline was verified to stimulate apoptosis and inhibit the growth, migration, and invasion of pancreatic cancer BxPC-3 cells. These effects may be associated with suppressed HSP90α expression, induced ERBB2 degradation, and subsequent inhibition of STAT3 and PI3K/Akt/mTOR pathway activation, as well as altered expression of multiple downstream proteins. **Conclusions:** This study demonstrates that dehydrocorydaline is the main active component of *Corydalis yanhusuo* W.T. Wang with anti-pancreatic cancer activity. Based on protein expression-level evidence, it may exert its effects by inhibiting HSP90α expression and inducing ERBB2 degradation, thereby affecting the PI3K/Akt/mTOR and STAT3 pathways, ultimately suppressing proliferation, migration, and invasion while promoting apoptosis in BxPC-3 cells. These findings justify further investigation of dehydrocorydaline as a potential treatment for pancreatic cancer.

## 1. Introduction

Globally, pancreatic cancer is the third most common cause of cancer-related death. With a five-year survival rate of only 13%—the lowest of all major cancers—the outlook for this disease is still dismal despite tremendous advancements in oncology [1]. By 2030, pancreatic cancer is expected to surpass colorectal cancer as the second most prevalent cause of cancer-related death due to its steadily rising incidence worldwide.

Pancreatic ductal adenocarcinoma (PDAC), despite being the most common histological subtype, still lacks a complete understanding of its etiology and pathogenesis. It has been reported that smoking [2,3], diabetes [4], and family history [5] may increase the risk of pancreatic cancer. In addition, genetic mutations, such as mutations in Kirsten rat sarcoma viral oncogene homolog (*KRAS*), Breast Cancer susceptibility gene 1/2 (*BRCA1/2*), Cyclin dependent kinase inhibitor 2A (*CDKN2A*), Tumor protein p53 (*TP53*), and SMAD Family member 4 (*SMAD4*)—are frequently observed in PDAC [6,7,8,9]. These mutations can drive oncogenesis by activating multiple signaling pathways, notably PI3K/Akt/mTOR, JAK/STAT3, and RAF/MEK/ERK [10,11,12,13].

The only potentially effective treatment for pancreatic cancer is still surgery [14]. Due to the aggressive nature of the tumor and substantial obstacles to early detection, over 80% of patients are diagnosed with locally advanced or metastatic disease. For these patients, systemic therapy remains the cornerstone of management. Although gemcitabine- or 5-fluorouracil-based regimens are widely used, their clinical efficacy remains limited. Therefore, identifying new therapeutic agents is of critical importance.

Natural products represent a significant source of anticancer drugs owing to their wide availability, multi-targeted mechanisms of action, and complex regulatory pathways [15,16,17,18,19]. *Corydalis yanhusuo* W.T. Wang, also known as Yuanhu, is the dried tuber of *Corydalis yanhusuo* from the poppy family [20]. In clinical practice, *Corydalis yanhusuo* is mainly used for analgesia [21,22,23], and its sedative, antiarrhythmic, antibacterial anti-inflammatory tonic effects have also received widespread attention [24,25,26,27]. Recently, growing interest has emerged regarding its potential as an anticancer agent.

According to recent studies, the main active ingredient extracted from *Corydalis yanhusuo* W.T. Wang has anti-tumor characteristics in a variety of cancers, including non-small cell lung cancer [28]. Dehydrocorydaline (DHC) induces tumor cell apoptosis by modulating the Bax/Bcl-2 ratio and activating caspase-3, -7, -8, and -9. Furthermore, DHC suppresses the proliferation and metastasis of melanoma and breast cancer cells, potentially through modulation of the MAPK signaling pathway and regulation of key proteins such as Cyclin-dependent kinase 1 (CDK1), Cyclin D1 (CCND1), Matrix metallopeptidase 2 (MMP2), and Matrix metallopeptidase 9 (MMP9) [29].

This study used BxPC-3 pancreatic cancer cells as the experimental model. The antitumor effects of the compound were systematically explored via multiple in vitro functional experiments including MTT assay, colony formation assay, transwell assay and Western blot, combined with bioinformatics analysis. This work may provide a theoretical foundation for the development of novel therapeutic strategies for pancreatic cancer.

## 2. Results

### 2.1. Treatment with the Main Active Ingredient of Corydalis yanhusuo W.T. Wang Concentration-Dependently Suppressed Proliferation and Clonogenicity, Induced Apoptosis, Attenuated Migration and Invasion in Pancreatic Cancer BxPC-3 Cells

**Figure 1:**
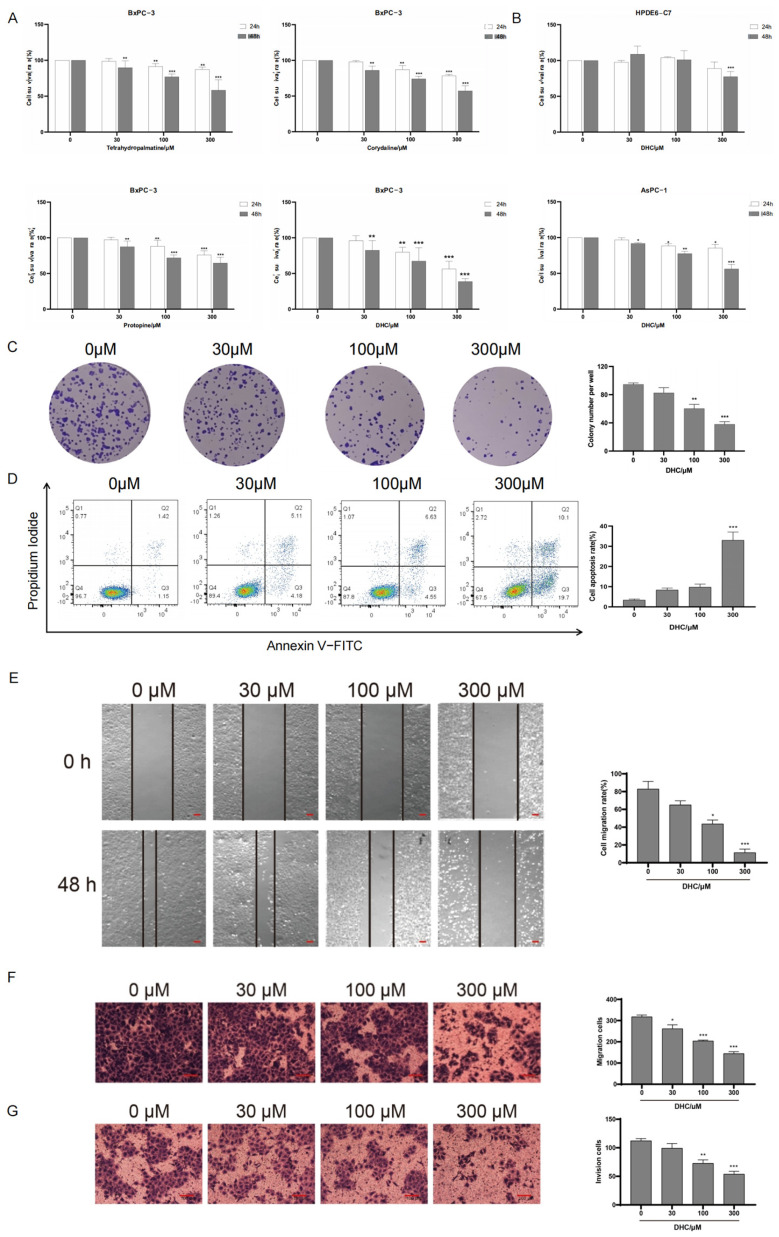
(**A**) The growth percentage of BxPC–3 cells exposed to varying tetrahydropalmatine, corydaline, protopine and DHC concentrations at various times. (**B**) The percentage of HPDE6–C7 and AsPC–1 cells that survived after being exposed to various DHC concentrations at various times. (**C**) The impact of pancreatic cancer cell treatment BxPC–3 with varying DHC concentrations for 48 h on the capacity to produce clones. (**D**) Flow cytometry investigation of the impact on apoptotic rate of treating pancreatic cancer cells BxPC–3 with varying DHC concentrations for 48 h. (**E**) Impact of varying DHC concentrations on BxPC–3 cells’ capacity to repair scratches. (**F**) The impact of varying DHC concentrations on BxPC–3 cells’ capacity to migrate. (**G**) The impact of varying DHC concentrations on BxPC–3 cells’ capacity for invasion. (Data: mean ± SEM; *n* = 3 biologically independent experiments; Scale: 100 μm; * *p* < 0.05 vs. 0 μmol/L Group; ** *p* < 0.01 vs. 0 μmol/L Group; *** *p* < 0.001 vs. 0 μmol/L Group).

To investigate the effect of *Corydalis yanhusuo* W.T. Wang on the survival rate of pancreatic cancer cells BxPC-3, we first selected four active components: tetrahydropalmatine, corydaline, protopine, DHC. The MTT assay was utilized to evaluate the inhibitory effects on BxPC-3 pancreatic cancer cells. Cells were treated with different concentrations of tetrahydropalmatine, corydaline, protopine, and DHC (0, 30, 100, 300 μmol/L) for 24 and 48 h. Results indicated DHC exhibited the most pronounced effect. At 100 μmol/L concentration after 24 h treatment, the survival rates of BxPC-3 pancreatic cancer cells were 80.65 ± 4.64% and 57.26 ± 3.24% at 48 h. At 300 μmol/L concentration after 24 h treatment, 48 h, the survival rates of pancreatic cancer cells BxPC-3 were 61.57 ± 2.23% and 32.94 ± 3.45%, respectively (Figure 1A). And its IC_50_ values were 372.8 μmol/L after 24 h treatment and 193.8 μmol/L after 48 h treatment. Consequently, dehydrocorydaline for 48 h was selected as the representative component of *Corydalis yanhusuo* W.T. Wang for further investigation. Concurrently, at concentrations of 30 μmol/L and 100 μmol/L, DHC exhibited no significant inhibitory effect on normal pancreatic cells HPDE6-C7 after 24 h and 48 h treatments, respectively. Following 48 h treatment with 300 μmol/L DHC, the survival rate of HPDE6-C7 cells was 80.59 ± 2.60%. In addition, DHC exhibits antiproliferative activity against AsPC-1 cells (Figure 1B).

A colony formation assay was used to assess the impact of DHC on the clonogenic ability of BxPC-3 cells. For 48 h,BxPC-3 were exposed to several doses of DHC (0, 30, 100, and 300 μmol/L). Compared with the control group, DHC reduced both the size and number of BxPC-3 cell colonies. These results indicated that DHC could inhibit the clonogenic ability of BxPC-3 cells (Figure 1C).

BxPC-3 cells were examined using flow cytometry to assess the effect of DHC on apoptosis. The results showed that DHC induced concentration-dependent apoptotic cell death, with maximal effect observed at 300 μmol/L after 48 h of treatment (Figure 1D).

The propensity of pancreatic cancer to metastasize contributes significantly to its high patient mortality. The migratory ability of human pancreatic cancer BxPC-3 cells was evaluated using both scratch wound and transwell assays to assess the impact of DHC on cell migration. After 48 h of treatment, DHC significantly reduced the wound-closing ability of BxPC-3 cells in a concentration-dependent manner compared with the control (Figure 1E). Furthermore, exposure to DHC (30, 100, and 300 μmol/L) for 48 h significantly suppressed BxPC-3 cell migration, with the inhibitory effect intensifying in a clear concentration-dependent manner (Figure 1F). Matrigel was applied to the upper transwell chamber to mimic the extracellular matrix (ECM). Tumor cells were then able to degrade this matrix by secreting matrix metalloproteinases (MMPs), thereby facilitating their migration through the chamber. Consequently, the impact of various DHC concentrations (0, 30, 100, and 300 μmol/L) on the invasive capacity of BxPC-3 cells was assessed using a transwell assay. The results showed that, compared with the control group, the number of BxPC-3 cells migrating to the lower chamber was significantly reduced after 48 h of treatment in a dose-dependent manner (Figure 1G). Collectively, these data indicate that DHC significantly impaired the migration and invasion of BxPC-3 cells.

### 2.2. Bioinformatics to Predict Key Targets and Signaling Pathways of Dehydrocorydaline for Pancreatic Cancer Treatment

**Figure 2:**
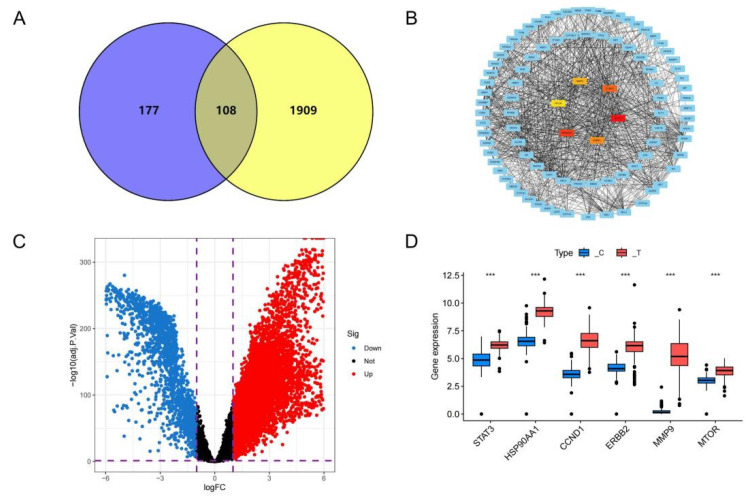
(**A**) A total of 285 drug targets for DHC and 2017 disease targets for pancreatic cancer were obtained through bioinformatics analysis, and the intersection of these two targets represented 108 efficacy targets for DHC treatment of pancreatic cancer. (**B**) Core gene PPI network screening map (based on Cytoscape). The red nodes in the middle are the top 6 core genes ranked by degree. The shade of red is positively correlated with the degree score (the darker the color, the higher the connectivity of the gene in the PPI network). (**C**) The TCGA-PAAD (The Cancer Genome Atlas-Pancreatic Adenocarcinoma) dataset was used to create a volcano chart of genes with different expressions. (**D**) Box plot of differential expression of core genes. (*** *p* < 0.001).

The canonical SMILES notation of DHC was retrieved from PubChem and used to query the SwissTargetPrediction, TargetNet, SuperPred, and SEA databases. After removing duplicates, this process yielded 285 unique DHC-associated targets. Similarly, pancreatic cancer (PC)-related targets were retrieved from the TTD, OMIM, GeneCards, and DisGeNET databases, resulting in 2017 unique entries following duplicate removal. The intersection of drug- and disease-associated targets identified 108 overlapping targets (Figure 2A), which are predicted to represent potential therapeutic targets of DHC in pancreatic cancer that warrant further investigation.

The 108 putative DHC targets were imported into the STRING database to generate a protein–protein interaction (PPI) network (interaction confidence score threshold: 0.4). The resulting PPI network—suggesting potential therapeutic targets of DHC in pancreatic cancer—was visualized using Cytoscape software version 3.10.3. (Figure 2B). The cytoHubba plugin was then applied to rank nodes based on degree centrality, leading to the identification of the top six hub targets: matrix metalloproteinase 9 (MMP9), heat shock protein 90α family class A member 1 (HSP90AA1), signal transducer and activator of transcription 3 (STAT3), cyclin D1 (CCND1), mechanistic target of rapamycin (MTOR), and receptor tyrosine-protein kinase erbB-2 (ERBB2). These in silico-predicted hub proteins may play important roles in the putative mechanism of DHC.

We leveraged The Cancer Genome Atlas-Pancreatic Adenocarcinoma (TCGA-PAAD) dataset to perform a differential expression analysis, the results of which are displayed in the volcano plot (Figure 2C). The expression patterns of the six high-degree targets were further profiled with box plots based on the differential expression analysis (Figure 2D). According to this bioinformatic analysis, the expression levels of these genes differed significantly between pancreatic cancer and normal tissues, suggesting a potential association with pancreatic cancer pathology. However, these findings are computational predictions and require experimental validation.

**Figure 3:**
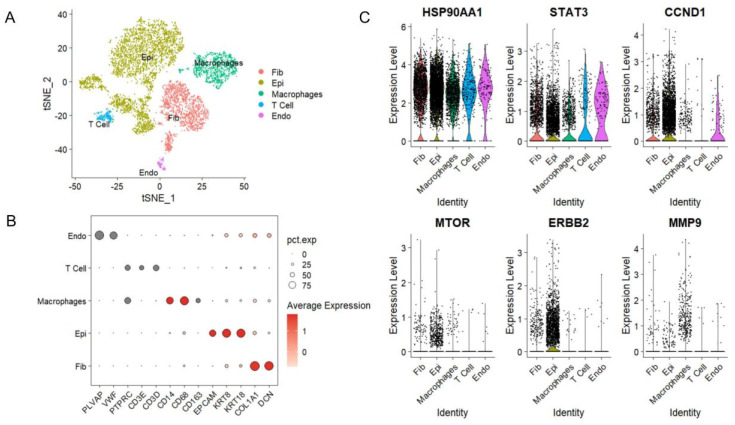
(**A**) Single–cell atlas of pancreatic cancer visualized by t–SNE. (**B**) Marker genes annotated in the pancreatic cancer single–cell atlas. (**C**) Violin plots showing the expression of six candidate genes in the pancreatic cancer single–cell atlas.

To explore the potential cellular distribution of the predicted core genes at single-cell resolution, we performed t-SNE visualization of pancreatic cancer single-cell samples to construct a comprehensive transcriptomic atlas (Figure 3A). Through subsequent cell-type annotation using specific marker genes, we identified distinct cellular populations (Figure 3B). In Figure 3B, the x-axis shows unique marker genes, the y-axis shows annotated cell types, the color intensity represents the average expression level of marker genes, and the circle size indicates the percentage of cells expressing each marker.

Violin plots were generated to profile the cell-type-specific expression patterns of six candidate genes (Figure 3C), with the x-axis representing different cell populations and the y-axis depicting expression levels. According to this single-cell transcriptomic analysis, *HSP90AA1*, *STAT3*, *CCND1*, *MTOR*, and *ERBB2* showed a tendency of specific distribution and expression in malignant epithelial cells, suggesting that these genes may be associated with malignant cell populations. In contrast, *MMP9* did not show such a trend; it appeared to be more loosely distributed and was predominantly expressed in macrophages.

**Figure 4:**
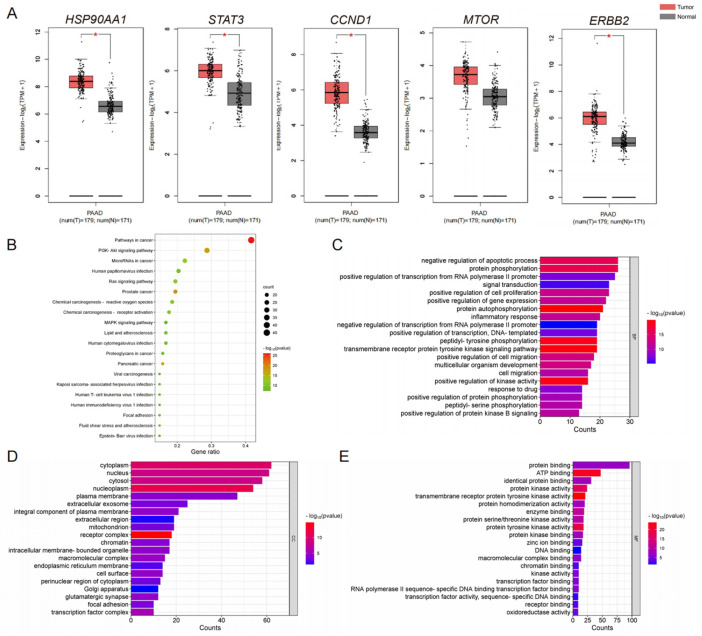
(**A**) Examination of how important targets are expressed differently in pancreatic cancer and healthy pancreas. (**B**) GO analysis of 108 protein targets for DHC treatment of PC. (**C**–**E**) KEGG analysis of 108 protein targets for DHC treatment of PC. (* *p* < 0.05).

The five key targets were submitted to the GEPIA 2 database to assess their mRNA expression. Except for *MTOR*, the expression of *HSP90AA1*, *STAT3*, *CCND1*, and *ERBB2* was markedly elevated in PC tissues as compared to normal controls, as seen in Figure 4A. These observed differences suggest a potential association with pancreatic cancer. We performed functional enrichment profiling on the 108 pharmacodynamic targets by interrogating the DAVID database for GO and KEGG analyses. The GO enrichment analysis indicated that the DHC targets were predicted to be enriched in biological processes such as protein phosphorylation, negative regulation of apoptosis, and regulation of transcription from RNA polymerase II promoter (Figure 4B). Separately, the KEGG enrichment analysis identified significant enrichment of the pharmacodynamic targets in pathways involved in cancer and the PI3K-Akt signaling axis (Figure 4C–E). A comparative analysis of the KEGG pathways revealed that the PI3K-Akt signaling pathway may be a key component of the broader cancer pathway. This pathway contains four key targets, namely *HSP90AA1*, *CCND1*, *MTOR* and *ERBB2*. These findings suggest that the PI3K-Akt signaling pathway may be a key pathway through which DHC may exert its anti-tumor effects in pancreatic cancer.

### 2.3. Dehydrocorydaline Suppresses the Growth and Metastasis of BxPC-3 Cells by Modulating the Activity of Key Regulatory Proteins

**Figure 5:**
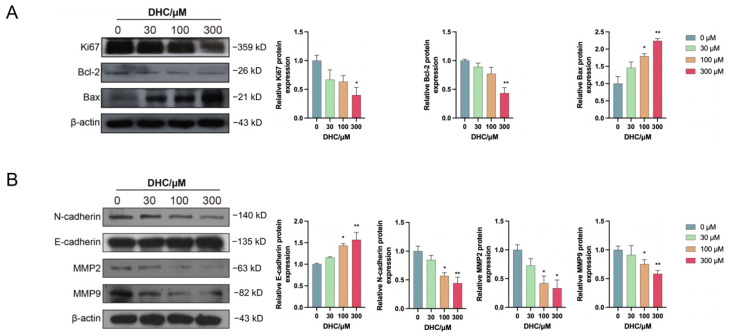
(**A**) DHC’s effects on BxPC-3 proliferation and apoptosis-related protein production. (**B**) DHC’s effects on the development of migration and invasion proteins in BxPC-3 cells. (Data: mean ± SEM; n = 3 biologically independent experiments; * *p* < 0.05; ** *p* < 0.01 vs. 0 μmol/L group).

To investigate the mechanisms through which DHC influences BxPC-3 cell proliferation and apoptosis, we assessed the expression of key signaling molecules involved in these processes and in metastasis via Western blot. DHC treatment significantly altered protein expression: it downregulated Ki67 and Bcl-2 while upregulating Bax (Figure 5A). Furthermore, DHC suppressed the expression of metastasis-associated proteins N-cadherin, MMP2, and MMP9, but enhanced E-cadherin levels (Figure 5B).

### 2.4. Validating the Anti-Pancreatic Cancer Mechanism of DHC: Key Targets and Pathways in BxPC-3 Cells

**Figure 6:**
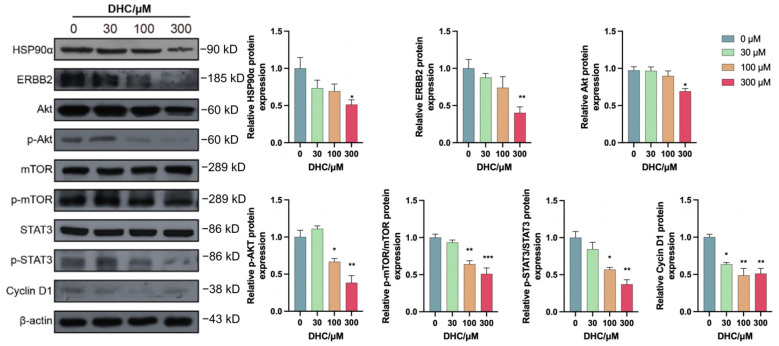
Effects of DHC on the expression of key targets and pathway-related proteins in BxPC-3 cells. (Data: mean ± SEM; n = 3 biologically independent experiments; * *p* < 0.05 vs. 0 μmol/L group; ** *p* < 0.01 vs. 0 μmol/L group; *** *p* < 0.001 vs. 0 μmol/L group).

To validate the predicted mechanism of DHC in pancreatic cancer, Western blot analysis was performed to evaluate its effects on key targets (HSP90α, STAT3, Cyclin D1, mTOR, ERBB2) and essential elements of the BxPC-3 cells’ PI3K/Akt signaling pathway. As shown in Figure 6, DHC treatment significantly downregulated the expression of multiple key proteins, including ERBB2, HSP90AA1, AKT, phosphorylated AKT (p-AKT), phosphorylated mTOR (p-mTOR), phosphorylated STAT3 (p-STAT3), and Cyclin D1. In summary, our data indicate that DHC exerts its antitumor effects by downregulating HSP90α and inhibiting both the ERBB2/PI3K/AKT/mTOR and STAT3 signaling axes. Mechanistically, this ultimately leads to increased apoptosis and decreased cellular proliferation and metastasis in pancreatic cancer.

## 3. Discussion

Previous studies have reported that the alkaloids of *Corydalis yanhusuo* W.T. Wang exhibit various pharmacological effects, including anti-tumor activity [21,23,27]. To explore their anti-tumor potential, we tested four representative alkaloids (tetrahydropalmatine, corydaline, protopine, and dehydrocorydaline) on BxPC-3 pancreatic cancer cells. DHC showed the strongest inhibition of cancer cell viability, with only minor effects on normal pancreatic cells.

We first gathered DHC targets and PC-related targets through database searches in order to examine the possible therapeutic targets and molecular processes of DHC in the treatment of pancreatic cancer. Through intersection analysis, we screened out 108 potential pharmacodynamic targets—i.e., key targets that may represent direct associations between DHC and pancreatic cancer. Based on PPI network analysis, six core targets were further identified from these 108 targets. To validate the disease relevance of these core targets, we analyzed clinical sample data (pancreatic cancer vs. normal pancreatic tissues). The results showed that, with the exception of MTOR, the other five core targets were significantly upregulated in pancreatic cancer tissues. Further single-cell transcriptome analysis revealed that five of these core targets were specifically highly expressed in pancreatic cancer malignant epithelial cells (the main tumor cell population), while only MMP9 did not exhibit this characteristic, suggesting a weak association with tumor cells. The PI3K/Akt signaling network, cancer-related pathways, protein phosphorylation, and the negative regulation of apoptosis were all strongly linked to the possible pharmacological targets, according to enrichment analysis. Cross-analysis confirmed that the PI3K/Akt signaling pathway is a common core pathway of these two pathways and contains four core targets. In order to further investigate the anti-tumor activity of DHC, it was discovered that DHC could inhibit the proliferation, metastasis, and induce the death of pancreatic cancer cells through scratch wound, transwell, and apoptosis. This suggests that this pathway may be the primary molecular pathway through which DHC exerts anti-pancreatic cancer effects.

The PI3K/Akt signaling pathway is aberrantly activated in many malignant tumors (including pancreatic cancer). Malignant tumors can be effectively treated by blocking its function. In addition to promoting apoptosis and cell cycle arrest, blocking the PI3K/Akt signaling pathway can prevent pancreatic cancer cells from proliferating, migrating, and invading [22,30]. Conversely, activation of the PI3K/Akt pathway is well established as a key driver of pancreatic cancer progression, facilitating proliferation, migration, and invasion while suppressing apoptosis [31]. The MTOR gene encodes the highly conserved serine/threonine protein kinase known as the mammalian target of rapamycin. Abnormal activation can lead to the occurrence and development of tumors [32]. MTOR can act as a downstream protein of the PI3K/Akt signaling pathway, promoting tumor growth, metastasis, and angiogenesis [33].

The *ERBB2*/*HER2* gene encodes human epidermal growth factor receptor 2 (HER2), a member of the EGFR family. Aberrantly expressed in various malignancies, ERBB2 binds to other EGFR family members to activate downstream STAT3 and PI3K/Akt pathways, promoting proliferation, differentiation, and survival, thereby driving cancer initiation and progression [34,35,36,37].

HSP90α, encoded by *HSP90AA1* is a highly conserved, ATP-dependent molecular chaperone that interacts with more than 200 client proteins. As such, it safeguards client protein stability and modulates their activation in various cancers, critically supporting tumorigenic processes including cell proliferation, survival, migration, and oncogenic signaling [38,39,40]. In addition, ERBB2 is one of the client proteins of heat shock protein 90 (HSP90). It has been reported that HSP90 inhibitor geldanamycin can effectively induce ubiquitination and proteasome degradation of ERBB2.

The protein encoded by the *STAT3* gene is signal transducer and activator of transcription 3, which is a member of the STAT protein family and is responsible for extracellular to intracellular signal transduction and transcriptional regulation. Studies have found that STAT3 is abnormally expressed and activated in a variety of cancers. When STAT3 is activated, it moves as a homodimer to the nucleus, where it controls downstream gene transcription, promoting the development and spread of tumors.

The protein encoded by *CCND1* gene is cyclin D1, and its mutation, amplification and overexpression can lead to cell cycle disorder [41].

This study suggests that DHC may coordinately affect HSP90α and ERBB2, which is associated with suppressed activation of the PI3K/Akt/mTOR and STAT3 signaling pathways. Consistent with these observations, DHC treatment correlates with downregulation of key proliferation (Ki67), invasion (MMP2 and MMP9), and migration—associated markers (increased E-cadherin and decreased N-cadherin expression). Furthermore, alterations in apoptotic regulators downstream of these pathways—specifically, increased expression of the pro-apoptotic gene Bax and decreased expression of the anti-apoptotic gene Bcl-2—are observed in parallel with DHC-induced apoptosis. These protein expression-based correlative findings point to a potential mechanism of action; however, causal relationships require functional validation in future studies.

It is worth noting that the role of MMP9 in cancer progression is not limited to its expression in tumor cells. Increasing evidence from single-cell transcriptomic analyses indicates that, within the tumor microenvironment (TME), MMP9 is predominantly expressed by tumor-associated macrophages (TAMs) rather than by malignant epithelial cells. This observation, however, does not contradict our finding that DHC reduces MMP9 protein levels in purified pancreatic cancer cell lines. Instead, it highlights a dual-source model of MMP9 function during tumor progression. In our study, the decrease in MMP9 following DHC treatment was detected specifically in BxPC-3 cells, demonstrating that pancreatic cancer cells intrinsically express MMP9 and that this expression is suppressed upon inhibition of the HSP90α/ERBB2/PI3K/Akt/mTOR/STAT3 axis. The observed downregulation of MMP2 and MMP9, along with the reduced N-cadherin and increased E-cadherin, is consistent with the role of tumor cell-derived MMP9 in promoting ECM degradation, invasion, and migration. Collectively, bulk transcriptomic and functional screening identify MMP9 as a hub target due to its overall pathophysiological importance, whereas single-cell data reveal its major cellular source in the TME. Both perspectives are complementary and together reinforce the therapeutic rationale for targeting upstream signaling pathways that control MMP9 expression in tumor cells themselves [42,43].

A similar distinction between transcriptional and post-translational regulation applies to our observations on the mTOR pathway. Analysis of the TCGA dataset revealed no significant difference in *MTOR* mRNA expression between pancreatic tumor and normal tissues. However, our Western blot results demonstrated a clear reduction in p-mTOR following DHC treatment. This apparent discordance is not contradictory but rather highlights the different layers of gene regulation governing mTOR activity. As a serine/threonine kinase, mTOR is primarily activated through post-translational phosphorylation events in response to upstream signals such as PI3K/Akt, rather than through transcriptional upregulation of the MTOR gene itself. Therefore, the absence of differential MTOR mRNA expression does not preclude constitutive pathway activation at the protein level. Importantly, DHC targets the HSP90α/ERBB2 axis, thereby suppressing the upstream signaling that drives mTOR phosphorylation. The observed reduction in p-mTOR without a corresponding change in *MTOR* mRNA indicates that DHC inhibits kinase activation rather than gene transcription. This distinction is critical for interpreting the mechanism of action of DHC and aligns with the general behavior of kinase-targeted inhibitors.

Although the DHC concentrations employed in this study appear relatively high compared with conventional targeted agents, they are pharmacologically justified. As shown in Figure 1B, DHC at these concentrations exhibited markedly lower cytotoxicity toward normal human pancreatic ductal epithelial cells (HPDE6-C7) than toward BxPC-3 cancer cells. This selective anticancer activity is mechanistically consistent with the dependence of cancer cells on the HSP90α/ERBB2/PI3K/Akt/mTOR/STAT3 axis, a dependency that is less pronounced in normal cells due to greater signaling redundancy and lower baseline pathway activation. Thus, a therapeutic window exists wherein DHC effectively targets pancreatic cancer cells while sparing normal cells, supporting the pharmacological relevance of the concentration range used.

Several limitations of this study should be acknowledged. First, all mechanistic findings are based on protein expression data obtained from Western blot analysis, which can reveal correlations but cannot establish causality. Functional validation—such as genetic knockdown/overexpression of HSP90α or ERBB2, pharmacological inhibition, or rescue experiments—is necessary to determine whether the observed effects are indeed mediated by the proposed pathways. Second, the experiments were conducted exclusively in BxPC-3 cells, a single pancreatic cancer cell line. Given the high heterogeneity of pancreatic cancer, the findings may not be generalizable to other cell lines with different genetic backgrounds (e.g., *KRAS* or p53 mutations). Third, all data were obtained from in vitro assays; in vivo studies using animal models are required to assess the efficacy and safety of DHC in a physiological context. Future work will focus on addressing these gaps through systematic functional experiments and validation in diverse cellular and animal models.

## 4. Materials and Methods

### 4.1. Cell Lines and Compound Characterization

The human normal pancreatic cell line HPDE6-C7, the human pancreatic cancer cell line BxPC-3, and the human metastatic pancreatic adenocarcinoma cell line AsPC-1 were used in this study. All cell lines were maintained in RPMI 1640 medium supplemented with 10% fetal bovine serum (FBS), 1% (*v*/*v*) glutamate (Glu). The cells were cultured at 37 °C in a humidified atmosphere containing 5% carbon dioxide (CO_2_).

Dehydrocorydaline (CAS No. 30045-16-0; purity ≥ 97.5% by HPLC) was obtained from Shanghai Yuanye Bio-Technology Co., Ltd. (Shanghai, China) (Cat. No. B21273).

### 4.2. MTT

Cells at the logarithmic growth phase were harvested, dissociated, and prepared into a single-cell suspension. After counting, the cells were seeded into 96-well plates at a density of 1000 cells per well and placed in an incubator for culture. Following complete cell attachment, the cells were divided into different groups according to the experimental design. The blank group received no culture medium or cells; the control group was treated with culture medium without drugs; and the experimental groups were treated with culture medium containing corresponding drug concentrations. All groups were further incubated. After the designated treatment period, 200 μL of 0.5 mg/mL MTT solution was added to each well, and the plates were incubated for an additional 4 h. Subsequently, 150 μL of DMSO was added to each well. After the purple-red crystals were completely dissolved, the absorbance (OD value) was measured at a wavelength of 490 nm, and the cell viability was calculated.

### 4.3. Cell Clone Formation Assay

Cells were seeded into 6-well plates and cultured in an incubator with gentle shaking. After a certain period of culture, the original medium was discarded, and both a negative control group and experimental groups were established. The negative control group received 2 mL of DHC-free medium, while the experimental groups were treated with 2 mL of medium containing 30, 100, and 300 μmol/L DHC, respectively. Following a 48 h treatment, the medium was discarded, and fresh drug-free medium was added to all groups. The culture was continued until cell clones formed. After clone formation, the original medium was removed, and the cells were gently washed with PBS. The cells were then fixed with methanol for 15 min, followed by staining with 0.1% crystal violet for 30 min. Excess crystal violet solution was removed by washing with PBS. After air drying, images were captured, and clones (defined as cell clusters containing more than 50 cells) were counted.

### 4.4. Flow Cytometry

Cells were seeded into culture dishes. When the cell density reached approximately 60%, treatment was initiated. A control group and experimental groups were established. The negative control group was treated with DHC-free medium, while the experimental groups were treated with medium containing DHC at final concentrations of 30, 100, and 300 μmol/L, respectively.

After 48 h of culture, the supernatant from each dish was collected, and the cell layers were gently washed with PBS. The cells were then trypsinized. Subsequently, the cells collected from the supernatant together with the digested cells were pelleted by centrifugation at 1500 rpm for 5 min and washed twice with PBS.

According to the experimental group assignments, the samples were incubated for 15 min at room temperature in the dark. After incubation, the samples were washed once, resuspended in 190 μL of binding buffer. After thorough mixing, the samples were analyzed for apoptosis using a flow cytometer.

### 4.5. Cell Scratch Wound Assay

Cells were seeded into 24-well plates. When cell density reached approximately 95%, a vertical scratch was gently made across each well using a 10 μL pipette tip in a straight line. The old culture medium was then aspirated, and the wells were gently rinsed with 1 mL of basal medium to remove cell debris. A control group and experimental groups were established, with three replicate wells per group. The control group received 2% serum medium without DHC, while the experimental groups were treated with 2% FBS medium containing DHC at final concentrations of 30, 100, and 300 μmol/L, respectively. Images of the scratches were captured at 0 and 48 h after scratching. The scratch area was quantified using ImageJ 2 software, and cell migration (or wound closure) rates were subsequently calculated.

### 4.6. Transwell Assay

Cells at the logarithmic growth phase were harvested and prepared into a single-cell suspension using 2% FBS medium. After counting, an appropriate number of cells were seeded into the upper transwell chamber. The final concentrations of DHC in the upper chamber were 0, 30, 100, and 300 μmol/L. Then, 600 μL of complete medium containing 10% FBS was added to the lower chamber. After 48 h of incubation, the transwell chambers were removed. Cells remaining on the upper surface of the membrane were gently removed using a cotton swab. The cells that had migrated to the lower surface were fixed with methanol for 20 min, followed by staining with 0.1% crystal violet for 30 min. Excess crystal violet was carefully washed off. After the chambers were air-dried, images were captured under a microscope in randomly selected fields, and the number of migrated cells was counted.

### 4.7. Transwell Invasion Assay

Matrigel was diluted with serum-free medium to a concentration appropriate for preparing 100 μL per well. The diluted Matrigel was added to the upper chamber of the transwell apparatus and then placed in a 37 °C incubator for 1–2 h to allow solidification. Subsequently, the procedures for cell seeding, drug treatment, cell fixation, staining, image capture, and cell counting were performed exactly as described for the transwell migration assay.

### 4.8. Target Screening and Validation Based on Bulk Transcriptome Data

The key target data were imported into the GEPIA2 (http://gepia2.cancer-pku.cn/) database to analyze the expression differences between pancreatic cancer tissues and normal tissues. Meanwhile, we performed differential expression analysis based on The Cancer Genome Atlas-Pancreatic Adenocarcinoma (TCGA-PAAD) dataset, with the log fold change (logFC) filter set to 0.8 and the adjusted *p*-value (adj. *p*-val) filter set to 0.05, yielding genes that are upregulated and downregulated in pancreatic cancer.

### 4.9. Construction of Pancreatic Cancer Single-Cell Atlas and Screening of Disease-Related Genes

Downstream analysis of single-cell RNA sequencing data was performed using the Seurat package in R. The data used were from GSE154763 in the GEO database. First, the data were screened through a rigorous quality control process: We retained genes expressed in at least five cells (min. cells = 5) and selected high-quality cells with a gene detection number (nFeature RNA) between 300 and 8000 and mitochondrial gene content less than 5% (percent.mt < 5). After quality control, we normalized the data and identified the top 2000 hypervariable genes for subsequent analysis. Next, data scaling was performed; after dimensionality reduction via PCA, the Harmony algorithm was used to correct for batch effects. Dimensionality reduction, clustering, and visualization were carried out based on the corrected Harmony embeddings: after evaluating the contribution of principal components, the top 20 dimensions were selected to construct graph for clustering, and UMAP and t-SNE coordinates were calculated to visualize clustering results. Finally, biological annotation of each cell cluster was performed by detecting the expression of known cell type-specific canonical marker genes.

### 4.10. GO Function and KEGG Pathway Enrichment Analysis Based on Drug Targets of Dehydrocorydaline

Gene Ontology (GO) and Kyoto Encyclopedia of Genes and Genomes (KEGG) pathway enrichment analyses were performed using the DAVID database. The potential targets of dehydrocorydaline for the treatment of pancreatic cancer were submitted to the database with the species set to “human”. Enrichment analysis was conducted, and significantly enriched terms were identified using a threshold of *p*-value < 0.05. GO enrichment analysis annotated the targets into three categories: Biological Process (BP), Cellular Component (CC), and Molecular Function (MF). KEGG pathway analysis was used to identify the major signaling pathways involving these target proteins. The results were visualized using the Weishengxin online platform.

### 4.11. Western Blot

Logarithmic-phase cells were detached with trypsin and seeded into culture plates. When the cells reached 60% confluency, the control group was treated with medium without DHC, and the experimental groups were treated with medium containing final concentrations of 30, 100, and 300 μmol/L DHC. After treatment, whole-cell proteins were extracted, and protein concentrations were determined with a standard curve generated for quantification.

Equal amounts of protein were separated by SDS-PAGE and transferred to PVDF membranes. Membranes were blocked and incubated with primary antibodies, followed by HRP-conjugated secondary antibodies. Protein bands were visualized using an ECL detection system.

Band intensities were quantified using ImageJ software. For each target protein, the raw grayscale value was first background-subtracted and then normalized to the loading control within the same lane. Normalized values were then used to calculate fold changes relative to the control group.

### 4.12. Statistical Analysis

Experiments were independently repeated at least three times using three independent biological replicates (n = 3). Data are shown as mean ± SEM. SPSS 26 and GraphPad Prism 10 were used for analyses and graphing. Group differences were assessed by one-way ANOVA. *p* < 0.05 was considered statistically significant.

## 5. Conclusions

As the main active ingredient of *Corydalis yanhusuo* W.T. Wang, DHC promotes apoptosis while inhibiting migration, proliferation, and invasion in BxPC-3 pancreatic cancer cells. Based on our protein expression data, this multi-faceted anti-tumor activity is associated with reduced HSP90α expression and decreased ERBB2 levels, accompanied by diminished activation of the PI3K/Akt/mTOR and STAT3 signaling pathways. Our findings offer valuable preliminary insights into the clinical translation of DHC and may inform the future selection of targeted therapeutic strategies for pancreatic cancer.

## Data Availability

The original contributions presented in this study are included in the article. Further inquiries can be directed to the corresponding authors.
